# Morphological and molecular characterizations of *Heterodera oryzae* in Korea

**DOI:** 10.21307/jofnem-2020-113

**Published:** 2020-11-06

**Authors:** Rose Mwesige, Eun-Hwa Kim, Eun-Hyung Park, Hyoung-Rai Ko

**Affiliations:** 1 National Agricultural Research Organization, Kachwekano ZARDI, P.O. Box 421, Kabale, Uganda; 2 Crop Protection Division, National Institute of Agricultural Sciences, Rural Development Administration, Wanju, 55365, Korea

**Keywords:** *COI*, *Heterodera oryzae*, Internal transcribed spacer, LSU rRNA, Morphology

## Abstract

Rice is one of the most important staple grains in Korea and the largest starch source in addition to its usefulness in the production of beverages. Under different areas and environments of production, various pests and diseases including soil-borne plant pathogens such as plant-parasitic nematodes can compromise rice productivity. In a survey to identify plant parasitic nematodes on rice, cyst nematodes were encountered in rice fields that required characterization and identification. Phylogenetic analysis of the LSU D2-D3 region and ITS region could not separate the studied species from *Heterodera elachista*. However, phylogenetic analysis of the *COI* gene of the mitochondrial DNA clearly separated *H. elachista* from the new species into two different clusters. Combining morphology and molecular diagnostics, the species was identified as *Heterodera oryzae* belonging to the ‘Cyperi’ group whose cysts are characterized by vulval cones that are ambifenestrate, underbridge present with bullae. Second-stage juveniles have three incisors in the lateral field with long tails and long hyaline region.

Rice [*Oryza sativa* L.] is a staple food for about 3.5 billion people in the world (Singh and Singh, 2017), with an annual production of about 480 metric tons of milled rice consumption (Muthayya et al., 2014). Rice contributes about 50% of dietary caloric supply and forms part of substantial protein intake for an estimate of 520 million people in Asia (Muthayya et al., 2014). In South Korea, rice is a staple grain and a largest starch source (Choi et al., 2016). In 2019, rice production in Korea was estimated at 3,744,450 metric tons from 730,000 ha area under production (USDA Foreign Agricultural Service, 2019). A big area of 729,586 ha was under paddy rice compared to 229 ha only for Upland rice production (USDA Foreign Agricultural Service, 2019). In addition to being a table food, rice is important in the production of beverages such as Soju and Makgeoli/raw rice wine and in baking industries (Choi et al., 2016). Under different areas and environments of production, rice is exposed to various pests and diseases including soil bone plant pathogens such as plant-parasitic nematodes. Plant-parasitic nematodes have been documented as an important constraint to rice production in various parts of the world and can cause an estimated yield loss of 20 to 90% depending on the nematode species present country, season, crop variety, cultivation practices, and soil conditions (Gilces et al., 2016). The most important nematode species in rice production include *Meloidogyne graminicola*, *Hirschmanniella oryzae*, *Pratylenchus zea*, *Aphelechoides besseyi*, *Ditylenchus angustus*, *Pratylenchus indicus*, and some cyst nematodes especially *Heterodera oryzicola* (Prasad et al., 1987; Jairajpuri, 2019).

Four species of cyst nematodes infect rice viz *Heterodera elachista*, *H. oryzicola*, *H. sacchari*, and *H. oryzae* (Nobbs et al., 1992). Although cyst nematodes have been reported in rice from other countries neighboring South Korea such as China and Japan; no practical studies have indicated their presence in South Korea though Choi et al. (1989) listed *H. elachista* and *H. oryzae* among the rice nematodes in the country.

In a survey to identify plant-parasitic nematodes on rice in South Korea, cyst nematodes were found in two rice fields in Dang-jin city and this required a detailed study of the samples in order to get accurate information on the nematode species. Nematode species identification is a requirement before designing and undertaking any management strategy in crop production. Species identification using morphological characters alone is very difficult and does not give correct results. A combination of both morphological and molecular approaches is very crucial for proper and accurate identification (Subbotin et al., 2015). The use of mitochondrial DNA cytochrome c oxidase subunit I (mt*COI*) gene, ITS region, and D2-D3 expansion segments of 28S rDNA region sequences are trending today in molecular and phylogenetic studies (Hajibabaei et al., 2007; Subbotin et al., 2007). The objectives of this study were to identify cyst nematode species associated with rice in South Korea, provide morphological and morphometrical information, and characterize the nematodes using DNA barcoding.

## Materials and methods

### Nematode sampling, extraction, and pure culture establishment

Soil samples were taken from two rice fields in Dang-jin city – South Korea using soil auger from about 15 cm in depth and placed in plastic bags. Samples were labeled as SG936 and SG153 (Table 1). Cysts were extracted from 100 cm^3^ of a soil sample using the sieving method (20 and 60 mesh). To establish pure cultures, second-stage juveniles (J2s) obtained from a single cyst were used to infect rice plants growing in pots in a plant growth room kept at 25°C. The plants were left to grow for five months before extracting cysts from pot soil. After extraction, cysts were collected with forceps into a watch glass containing tap water using a stereomicroscope (MZ12; Leica, Wetzlar, Germany) and were kept at 4°C until further use.

### Morphological and morphometric traits for cysts and J2s

In total, 13 cysts for SG936 and 25 cysts for SG153 were used for full body measurement, vulval cone making, and J2s collection. Full shapes for cysts were captured to measure their length and width.

Vulval cones were cut under a stereomicroscope, transferred to glycerin on slide glasses for microscopic examination. Important characters for distinguishing cyst nematode species such as the underbridge length, presence of bullae, fenestral length, semifenestral width, and vulval slit length were observed under a light microscope, imaged and measured (Sekimoto et al., 2017).

Important taxonomic diagnostic characters for J2s were obtained by temporarily mounting juveniles on slide glasses for examination under a light microscope (DM5000; Leica, Wetzlar, Germany). An automatic camera attached to a LEICA DM5000 compound microscope was used to capture images for measurements. J2s measurements were made for body length, middle body width, stylet length, tail length, hyaline tail length, and excretory pore distance from the anterior part of the body. Measurements were recorded in µm, arithmetic mean, standard deviation, and measurement ranges obtained. Nematodes were identified morphologically based on *Heterodera sp.* identification key authored by Subbotin et al. (2010).

### Molecular characterizations

#### DNA extraction

To extract DNA, a single cyst was transferred to a glass slide on a small drop of distilled water, opened and its contents crushed using a filter paper chip (2 mm × 2 mm) and forceps. Using forceps, the chip having crushed eggs and J2s was transferred into a PCR tube containing 30 µl lysis buffer (autoclaved triple distilled water, 1 M Tris-HCl, 10% Triton-X 100, 100 µg/ml Proteinase K, 2 M KCl, 1 M MgCl_2_) for extracting nematode DNA (Iwahori et al., 2000). The tubes were then incubated in a PCR machine (PTC-200; MJ Research, Alameda, CA, USA) at 60°C for 1 hr and 94°C for 10 min. The DNA was stored at −20°C for later use in PCR reactions.

#### PCR amplification

##### ITS region

The internal transcribed spacer (ITS) region was amplified using 0.5 µl each of primers TW81 [5′-GTTTCCGTAGGTGAACCTGC-3′] and AB28 [5′-ATTGCTTAAGTTCAGCGGGT-3′] (Joyce et al., 1994). In a PCR tube containing 15 µl of PCR mixture ready to use (Ready-2x-Go with Taqplus; Nanohelix^TM^, Daejeon, Korea) was added 2 µl of nematode DNA extract, 32 µl of triple distilled water, and 0.5 µl of each primer making a total volume of 50 µl. Cycling included pre-denaturation step at 94°C for 5 min followed by 40 cycles of 94°C for 1 min, 57°C for 1 min, and 72°C for 2 min, and finished with one cycle at 72°C for 10 min.

##### 28S large ribosomal subunit D2-D3 expansion segment

Primers D2A [5′-ACAAGTACCGTAGGGAAAGTT-3′] and D3B [5′-TCGGAAGGAACCAGCTACTA-3′] (De Ley et al., 1999) were used to amplify the region. PCR conditions were pre-denaturation at 94°C for 6 min followed by 40 cycles of 94°C for 1 min, 57°C for 1 min, and 72°C for 1 min and finished with one cycle at 72°C for 6 min.

##### *COI* gene of the mtDNA

A primer set of JB3 (5′-TTTTTTGGGCATCCTGAGGTTTAT-3′) and JB5 (5′-AGCACCTAAACTTAAAACATAATGAAAATG-3′) (Derycke et al., 2005) was used in the PCR reaction. PCR conditions were pre-denaturation step at 94°C for 4 min followed by 40 cycles of 94°C for 30 sec, 57°C for 30 sec, and 72°C for 45 sec and finished with one cycle at 72°C for 5 min.

PCR products were analyzed by electrophoresis on 1% agarose gel with 1X TAE buffer for 25 min. The gels were visualized using UV transilluminator (UVCI-1100; Major science, New Taipei City, Taiwan).

#### Phylogenetic analysis

After amplification, PCR products were purified using PCR purification kit following the manufacturer’s manual (NucleoSpin Gel and PCR clean up; Macherey-Nagel, Duren, Germany). Purified DNA was sent for sequence analysis at GenoTech Corporation (Daejeon, Korea). Obtained sequences were edited using EditSeq application to remove low quality bases and assembled in SeqMan computer program. Assembled sequences were posted in National Center for Biotechnology Information (NCBI) to obtain similarity match with other *Heterodera* species whose sequence nucleotides are already in the Gene Bank. Sequences of ITS rDNA, 28S, and *COI* genes for *Heterodera* spp. registered in NCBI were obtained and merged with our sample sequences of ITS rDNA, 28S, and *COI*, respectively. With the help of previous studies, an outgroup taxon for each data set was obtained. *Rotylenchus urmiaensis* and *Cryphodera brinkmani* were used as outgroups for *COI*, ITS, and 28S data sets, respectively (Madani et al., 2018). The merged sequences were aligned using ClustalX 1.83 program and Bayesian analysis of the sequence datasets performed using MrBayes ver. 3.2.6. The general time reversible substitution model (GTR + I + G) was used for this analysis and ran with four chains for 1 × 10^6^ generations. MCMC (Markov Chain Monte Carlo) method was used to estimate the posterior probability of phylogenetic tree and a consensus tree was generated with a 50% majority rule. The trees were visualized and edited using Dendroscope version 3.5.7 (Daniel and Celine, 2012).

## Results

### Morphological and morphometrical traits

**Figure 1:**
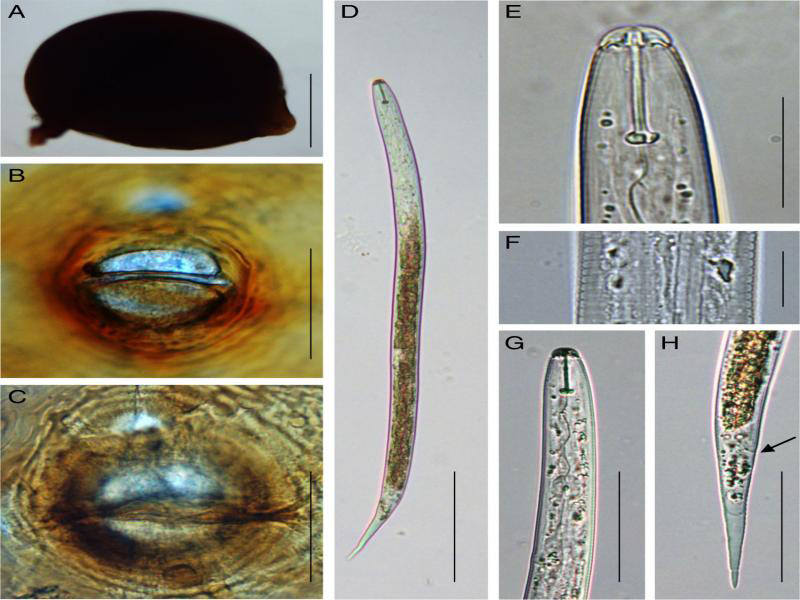
Morphological characteristics of cyst, vulval cone, and second-stage juvenile of *Heterodera* sp. (SG936). A-C: Cysts, (A) Lemon-shaped cyst (B) Anterior view (C) Underbridge level view, D-H: Second-stage juveniles (J2), (D) Entire J2 body (E) Stylet (F) Lateral field (G) Pharyngeal region (H) Tail region (black arrow: anus). *Scale bars: A = 100 μm, B = 50 μm, C = 50 μm, D = 100 μm, E = 20 μm, F = 10 μm, G = 50 μm, and H = 50 μm.

**Figure 2:**
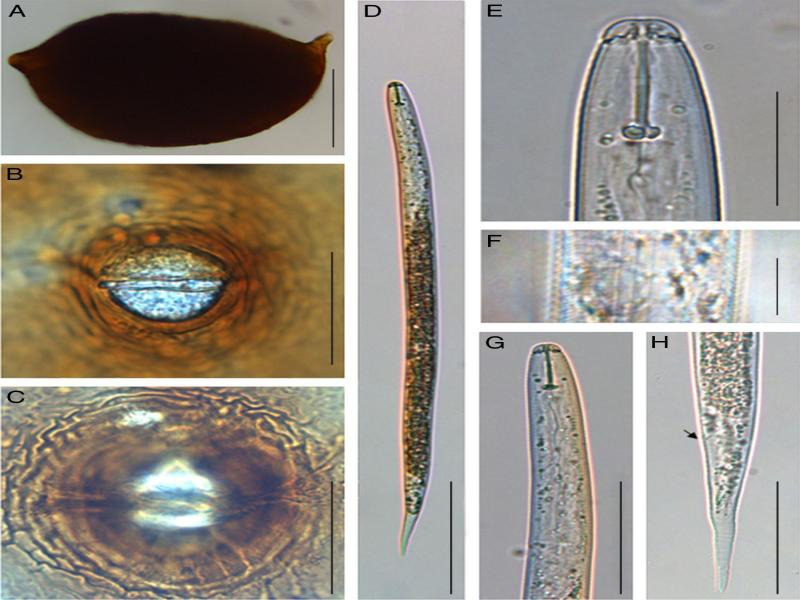
Morphological characteristics of cyst, vulval cone, and second-stage juvenile of *Heterodera* sp. (SG153). A-C: Cysts, (A) Lemon-shaped cyst (B) Anterior view (C) Underbridge level view, D-H: Second-stage juveniles (J2), (D) Entire J2 body (E) Stylet (F) Lateral field (G) Pharyngeal region (H) Tail region (black arrow: anus). *Scale bars: A = 100 μm, B = 50 μm, C = 50 μm, D = 100 μm, E = 20 μm, F = 10 μm, G = 50 μm, and H = 50 μm.

Morphological characters and morphometric features of J2s (stylet length, body length, tail length, and number of lateral lines) together with cysts and their vulval cone features were examined and measured for species identification. The color of cysts varied from light to dark brown having either oval or lemon shape. Vulval cones were ambifenestrate with bullae and a strong underbridge (Figs. 1, 2). Second-stage juveniles had a strong stylet with an average length of 21 µm. The body length ranged from 414 to 478 µm and 433 to 495 µm for SG936 and SG153, respectively (Table 2). The juveniles for the two samples had three incisors in the lateral field (Figs. 1F, 2F).

**Table 1.** Nematode samples used in this study.

Population code	Collection date	Host plant	Coordinates
SG936	July 27, 2016	Rice	36.902188, 126.661624
SG153	August 16, 2017	Rice	36.901005, 126.671008

**Table 2.** Morphometrics of second-stage juveniles and cysts for the *Heterodera* sp. of two populations SG153 and SG 936 as compared to *H. oryzae* and *H. elachista* in previous studies.

Population	SG936, South Korea, This study	SG153, South Korea, This study	*H. oryzae,* Côte d’Ivoire, Nobbs et al. (1992)	*H. elachista*, Japan, Nobbs et al. (1992)	*H. elachista*, Iran, Tanha Maafi et al. (2004)
Cysts (*n*)	13	25	50	50	39
Length (excl. neck)	498.9 ± 96.4 (307.0-617.2)	523.3 ± 54.5 (438.9-643.3)	714.8 ± 71.5 (442-999)	446 ± 30.3 (328-557)	431 ± 48 (340-586)
Width	429.3 ± 97.3 (251.2-550.1)	360.2 ± 47.2 (288.3-447.8)	426.6 ± 53.8 (229-655)	322.6 ± 26.0 (229-449)	311 ± 48 (225-540)
Length/width	1.2 ± 0.1 (1.0-1.4)	1.5 ± 0.2 (1.3-1.8)	1.71 ± 0.12 (1.1-2.3)	1.4 ± 0.1 (1.1-1.9)	1.4 ± 0.2 (1.1-1.9)
Vulval areas (*n*)	11	16	10	10	10
Fenestral length	36.5 ± 4.7 (28.2-40.9)	34.2 ± 4.9 (24.8-43.6)	38.8 ± 1.85 (32-46)	29.3 ± 2.0 (23.36)	40 ± 6.5 (30-50)
Semifenestral width	34.8 ± 4.4 (29.3-44.2)	35.7 ± 4.4 (25.9-41.0)	51.6 ± 5.20 (36-69)	32.0 ± 2.5 (26-39)	31.2 ± 4.7 (26-42)
Vulval slit length	33.1 ± 5.0 (27.6-45.6)	34.3 ± 3.8 (27.8-41.3)	50.7 ± 4.0 (41-65)	37.5 ± 2.8 (26-46)	40 ± 3.8 (32-45)
Vulval bridge width	9.0 ± 4.5 (4.4-17.9)	7.2 ± 3.3 (4.0-15.0)	12.14 ± 1.9 (6-16)	5.8 ± 2.0 (2-16)	–
Underbridge length	80.1 ± 7.2 (70.8-96.4)	83.0 ± 7.9 (63.4-95.3)	112.5 ± 9.0 (87-153)	72.0 ± 2.9 (65-78)	85
Vulva to anus distance	34.9 ± 3.0 (30.5-41.6)	36.6 ± 5.9 (30.1-50.3)	–	–	–
J2 (*n*)	11	11	20	20	10
*L*	450.6 ± 16.6 (414.7-478.6)	472.4 ± 19.3 (433.1-495.1)	554 ± 9.4 (523-589)	402 ± 10.0 (377-450)	391 ± 11 (372-410)
*a*	23.4 ± 0.7 (22.4-24.6)	23.3 ± 1.6 (20.1-25.9)	34.0 ± 1.03 (30.1-39.3)	24.6 ± 0.9 (21.3-28.1)	22.5 ± 1.0 (20.8-24)
*b*	–	–			
*c*	6.8 ± 0.3 (6.3-7.4)	6.8 ± 0.3 (6.3-7.2)	9.4 ± 0.3 (8.6-10.7)	7.0 ± 0.4 (5.4-8.4)	6.7 ± 0.3 (5.9-7.2)
Stylet (S)	21.1 ± 0.5 (20.4-22.4)	21.1 ± 0.8 (19.5-22.6)	20.5 ± 1.15 (16-25)	19.2 ± 0.8 (16-21)	20 ± 0.7 (18-21)
Labial region height	4.7 ± 0.5 (3.8-5.4)	4.7 ± 0.3 (4.3-5.3)	–	–	3
Labial region diam.	9.0 ± 0.7 (7.8-10.1)	9.1 ± 0.3 (8.7-9.6)	–	–	7.8 ± 0.4 (7-8)
DGO	7.3 ± 0.7 (6.5-8.4)	6.0 ± 0.8 (4.9-7.7)	–	–	5.2 ± 0.8 (4-7)
Anterior end to					
Excretory pore	97.5 ± 4.8 (87.5-103.0)	99.3 ± 6.6 (84.8-110.8)	–	–	84 ± 2.8 (79-88)
Median bulb valve (MB)	65.4 ± 2.7 (62.1-72.6)	64.4 ± 2.2 (60.6-68.0)	–	–	59 ± 3.6 (52-67)
Body diam. at					
Mid-body	19.2 ± 0.8 (18.1-20.7)	20.4 ± 1.9 (18.2-24.3)	16.3 ± 0.43 (15-18)	–	17.4 ± 0.8 (16-19)
Anus (BDA)	12.6 ± 0.9 (11.7-14.5)	13.3 ± 0.8 (12.0-14.6)	12.3 ± 0.72 (10-15)	11.4 ± 0.8 (10-13)	10.2 ± 0.6 (9-11)
Hyaline region (H)	36.9 ± 3.2 (29.3-41.0)	35.8 ± 3.4 (31.6-42.4)	32.8 ± 1.3 (26-44)	–	32 ± 3.0 (25-39)
Tail length (Tl)	66.9 ± 3.8 (60.5-73.4)	69.6 ± 4.7 (60.6-78.9)	58.8 ± 2.25 (49-66)	57.8 ± 2.9 (47-70)	–
H/S	1.7 ± 0.1 (1.4-1.9)	1.7 ± 0.2 (1.4-2.0)	1.6 ± 0.11 (1.4-2.2)	–	1.6 ± 0.1 (1.3-1.9)
L/MB	6.9 ± 0.2 (6.6-7.2)	7.3 ± 0.3 (6.9-8.1)	–	–	6.7 ± 0.4 (5.6-7.4)
Tl/H	1.8 ± 0.2 (1.6-2.5)	2.0 ± 0.1 (1.7-2.2)	–	–	–

### Molecular and phylogenetic analysis

**Figure 3:**
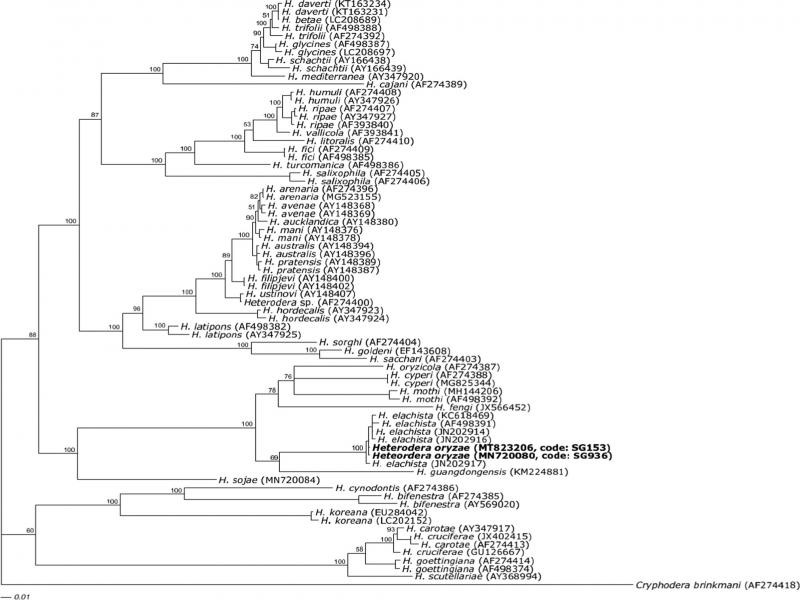
PCR results for ITS region, 28S D2-D3, and *COI* gene of SG153 and SG936 nematode populations with their respective universal primers using 100 bp DNA ladder.

ITS region, LSU D2-D3 (28S) segments, and *COI* gene of the mtDNA were amplified as indicated in methodology section. DNA fragment lengths after amplification showed (n = 6); 1000 bp for ITS region, 750 bp for 28S D2-D3, and 500 bp for *COI* gene, respectively (Fig. 3). Amplified DNA of ITS region, 28S region, and *COI* for SG936 and SG156 were sequenced. Reads for both nematode populations were between 956 and 979 bp, 751 and 759 bp, and 416 and 449 bp for ITS, 28S D2-D3, and *COI*, respectively (n = 6). Shorter sequences were due to some chromatographs having low quality signals at their beginning or ending. Bases that were not clear were trimmed to make sure that only high quality data were used.

Similarity search of the ITS sequences obtained from our study population revealed higher similarities with *H. elachista* compared to other *Heterodera* spp. Percentage identity with various populations of *H. elachista* ranged from 97.99 to 99.89%. Highest identity of 99.9% (947 and 960/1074) was with *H. elachista*, represented by GeneBank accession number JN202914 a nematode species isolated from rice in Hunan province in China.

Similarity search of the 28S sequences obtained in our study in NCBI GeneBank database ranged from 99.73 to 100% identity with *Heterodera elachista.* SG936 and SG153 LSU D2-D3 segments revealed a 100% (701 and 752/782) identity to *H. elachista*, GeneBank accession number MH767024 a nematode species isolated from corn field in Liaoning province in China.

A BLASTn search with the studied nematode sequences on the mt*COI* gene indicated higher similarity with *H. elachista* compared to other *Heterodera* species. A 97.73% (421 and 416/454) identity was revealed with *H. elachista*; GeneBank accession number MH144207.

**Figure 4:**
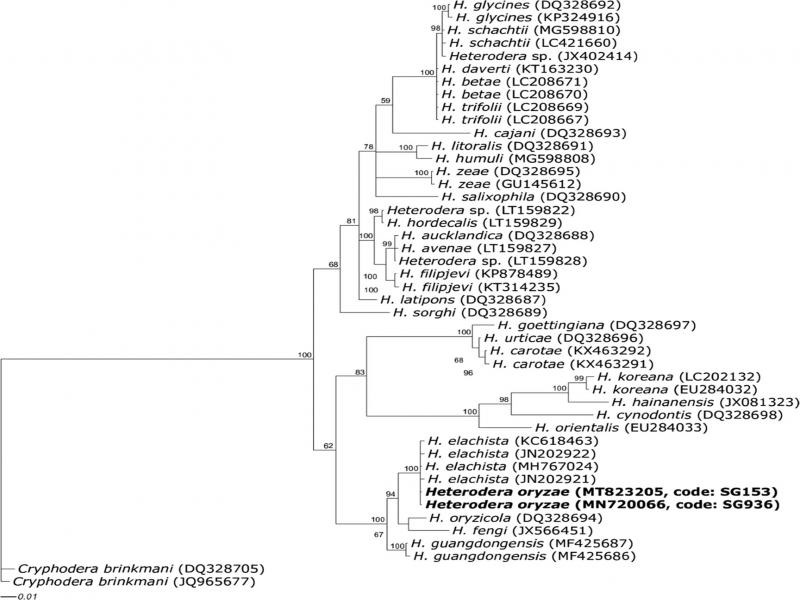
Phylogenetic relationship between two *Heterodera* populations (SG936, SG153) with selected representative sequences from many of the world known *Heterodera* species as inferred from Bayesian analysis of the internal transcribed spacer (ITS) sequence dataset. The newly obtained sequences were in bold. Posterior probability values more than 50% are given for appropriate clade.

**Figure 5:**
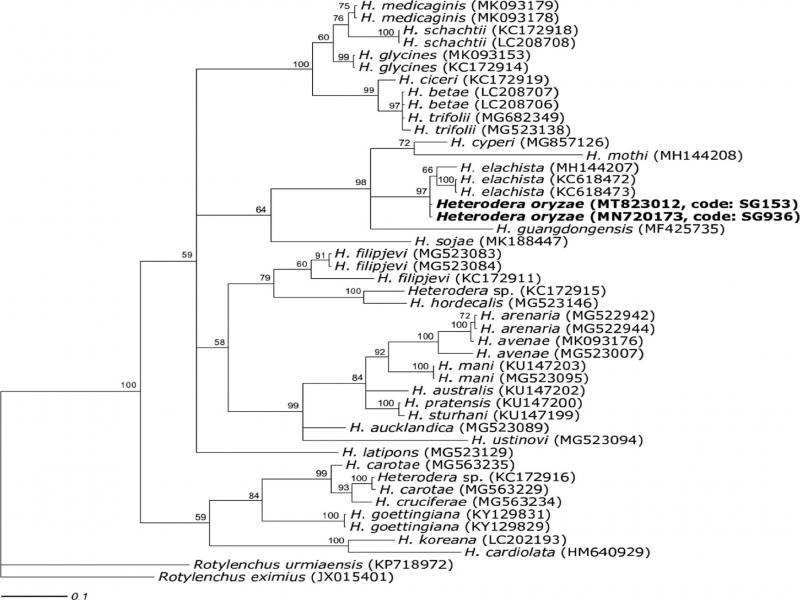
Phylogenetic relationship between two *Heterodera* populations (SG936, SG153) with selected representative sequences from many of the world known *Heterodera* species as inferred from Bayesian analysis of the cytochrome c oxidase subunit I of mitochondrial DNA sequence dataset. The newly obtained sequences were in bold. Posterior probability values more than 50% are given for appropriate clade.

Phylogenetic relationships of the studied species are indicated in Figures 3-5. Figure 3 shows a phylogenetic tree based on the ITS region data set of *Heterodera* species. The results showed that SG936 and SG153 belong to ‘Cyperi’ group and were very similar to *H. elachista.* Intraspecific variation of ITS region between SG936, SG153 populations, and *H. elachista* sequence data (JN202914) was 0.0%. A 0.3% intraspecific variation was obtained when five sequences of *H. elachista* (JN202914, AF498391, KC618469, JN202916, and JN202917) were used to compute intraspecific variation with SG936; SG153 sequences. Interspecific variation between SG936, SG153 populations and closely related species, *H. elachista* (JN202914, AF498391, KC618469, JN202916, JN202917), *H. oryzicola* (AF274387), *H. cyperi* (AF274388, MG825344), *H. guangdongensis* (KM224881), *H. mothi* (MH144206), and *H. fengi* (JX566452) were 0.4, 13.9, 14.7, 15.2, 17.3, and 17.4%, respectively.

The phylogenetic tree of the 28S region dataset of *Heterodera* species is shown in Figure 4. The results showed that SG936 and SG153 belong to ‘Cyperi’ group clade. Intraspecific 28S sequence variation between SG936, SG153, and some *H. elachista* 28S sequences (KC618463, JN202922, MH767024, and JN202921) was 0.2%. Interspecific 28S sequence variation between SG936, SG153 populations and other group species; *H. elachista* (KC618463, JN202922, MH767024, and JN20292921), *H. oryzicola* (DQ328696), *H. guangdongensis* (MF425686, MF425687), and *H. fengi* (JX566451) were 0.1, 3.1, 3.3, and 4.2%, respectively. According to this analysis, SG936 and SG153 were closely related to *H. elachista.*

The *COI* sequence dataset consisted of 44 sequences of *Heterodera* species together with the obtained sequences of SG153 and SG936. The phylogenetic tree as inferred from Bayesian analysis of *COI* gene sequence dataset is shown in Figure 5. The result showed that SG153 and SG936 nematode species belong to ‘Cyperi’ group clade. Intraspecific *COI* sequence variation between SG153, SG936, and *H. elachista* previous studies sequences was 2.4%, whereas interspecific *COI* sequence variation between SG153, SG936, and closely related species; *Heterodera elachista* (MH144207, KC618472, and KC618473), *H. guangdongesis* (MF425735), *H. cyperi* (MG857126), and *H. monthi* (MH144208) were 2.7, 14.7, 12.3, and 20.4%, respectively. From the results of *COI* sequences, we concluded that SG936 and SG153 populations were not *H. elachista*, but are just closely related species. Combining morphological and molecular diagnostics, SG936 and SG153 samples were identified as *H. oryzae*. This was the first time to study molecular characteristics of *H. oryzae*.

## Discussion

Using identification key by Subbotin et al. (2010) for *Heterodera* sp, the studied nematode populations were identified as *Heterodera oryzae.* Previous studies indicate that *H. oryzae* is closely related to *H. elachista*, a rice cyst nematode identified in Japan (Oshima, 1974; Nobbs et al., 1992). In this study, some characters that can separate *Heterodera oryzae* from *Heterodera elachista* were observed. Our sample consisted of large cysts with a rounded semifenestra; well-developed underbridge without finger like projections and bullae present. Some of these characteristics can be used to distinguish our species from *H. elachista* that is described as smaller cysts with a fragile and thinner underbridge tending to be absent in old cysts (Nobbs et al., 1992; Subbotin et al., 2010). *Heterodera elachista* and *Heterodera oryzae* belong to ‘Cyperi’ group whose J2s have three incisors in the lateral field, vulva cones have ambifenestrate fenestration type, weak or absent underbridge with bullae present or absent (Subbotin et al., 2010; De Luca et al., 2013). According to Nobbs et al. (1992), *H. elachista* second-stage juvenile stylets are shorter 19.2 µm compared to 20.5 µm of *H. oryzae*. The average stylet length of our sample species is 21.1 µm and is similar to the average stylet length of 21 µm for *H. oryzae* as published by Taylor (1978) and Subbotin et al. (2010). The studied samples J2s anterior end to excretory pore, hyaline length, and tail length were slightly bigger as compared to those reported by Nobbs et al. (1992) for *Heterodera oryzae*. The hyaline tail length of SG936, SG153 was 37 and 36 µm, respectively, almost the same with that of *H. oryzae* (38 µm) described by Taylor (1978). The differences that were observed in cysts, vulval cone, and J2 measurements in this study fall between the maximum and minimum values reported by other authors. Scan electron microscopy studies have been used with high reliance on the labial region to distinguish four cyst rice nematodes; *H*. *elachista*, *H. oryzicola*, *H. oryzae*, and *H. sacharri* (Nobbs et al., 1992). However, it is important to note that sole dependence on morphological characters to distinguish nematode species is very difficult and sometimes gives inconclusive results.

DNA barcoding has increasingly become common for efficiency and accuracy in nematode species identification (Amiri et al., 2003). The 28S, ITS, *COI* sequences of *H. oryzae* were obtained for the first time in this study. Species identification based on phylogenetic studies of the ITS and 28S region sequences could not separate our studied species from *H. elachista* because both markers showed limited interspecific variation thus were not useful for identifying these species. This confirmed the conclusions of other scientists that *H. elachista* and *H. oryzae* were closely related. However, phylogenetic studies of *COI* gene sequences clearly distinguished our species from *H. elachista* into distinct individual clusters of sequences with bootstrap of 97% in the phylogenetic tree. Our results are supported by a study of Vovlas et al. (2015) where *COI* gene of mitochorial DNA efficiently discriminated closely related *Heterodera* spp.; *H. daverti* from *H. trifolii* that could not be differentiated by either ITS or D2-D3 expansion segments of 28S rDNA.

Molecular analysis of mtDNA is very important to speed up and simplify the identification of animals such nematodes (Herbert et al., 2003a, 2003b; Lambshead, 2004). This is because the mtDNA is less exposed to recombination, lacks introns, and has a relatively high mutation rate compared to nuclear genome (Saccone et al., 1999). The features of mtDNA bring about a significant variation in mtDNA sequences between species than within species (Elsasser et al., 2009), facilitating phylogeny construction to address confusion in species boundaries and population variations (Blok and Powers, 2009).

The morphology of second stage juveniles and cysts and molecular analysis of *COI* gene of the mitochondrial DNA revealed the species identity as *Heterodera oryzae*. Detection of *H. oryzae* in rice fields of South Korea forms the first record of occurrence of this species in the country. Further studies are needed to provide evidence that the presence of *H. oryzae* in rice fields can result in economic losses in South Korean rice varieties. Additionally, scanning electron microscope studies on the species should be done to further characterize the species and supplement the above results.
